# Liver Lobe Torsion Following an Enterotomy for an Obstructive Foreign Body in a Puppy

**DOI:** 10.1002/vms3.70292

**Published:** 2025-03-29

**Authors:** Elizabeth C. Waskover, Jennifer Truong, Lindsay Phillips

**Affiliations:** ^1^ Wheat Ridge Animal Hospital, Denver Wheat Ridge Colorado USA

**Keywords:** Complications, Foreign body, Postoperative management, Surgery

## Abstract

A 6‐month‐old male intact Bernese Mountain Dog presented for lethargy and acute vomiting after known foreign body ingestion. The patient was diagnosed with an obstructive foreign body and underwent an exploratory laparotomy with an enterotomy. Twelve hours postoperative, he was diagnosed with a haemoabdomen. A second exploratory laparotomy was performed, and a left lateral and left medial liver lobe torsion was diagnosed. A left lateral liver lobectomy was performed. The left lateral liver lobe was submitted for histopathology and was consistent with acute torsion. This draws interest to the case as the patient is a juvenile dog and there was involvement of two liver lobes which is rare. The cause of liver lobe torsion is unknown in this case. Liver lobe torsion is rare and has never been reported in a young dog as an immediate postoperative complication.

## Graphical abstract

Liver lobe torsion is rare in canine patients, with more than one lobe affected being even more scarce. Liver lobe torsion of one or more lobes was found as an immediate postoperative complication for an exploratory laparotomy with an enterotomy performed in a juvenile dog.

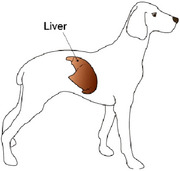

**Abbreviations**
aFAST: abdominal focused assessment with sonography for trauma
CBC: complete blood count
CRI: constant rate infusion
ECG: electrocardiogram
GDV: gastric dilation and volvulus
h: hour
ICU: intensive care unit
IV: intravenous
IVC: intravenous catheter
IVF: intravenous fluids
kg: kilogram
LLT: liver lobe torsion
LRS: lactated ringer's solution
mg: milligram
mg/dL: milligram per decilitre
min: minute
mL: millilitre
mmol/L: millimole per litre
PCV/TS: peripheral cell volume/ total solids
PO: by mouth
POCUS: point of care ultrasound
PRN: pro re nata (as needed)
Q12h: every 12 hours
Q6h: every 6 hours
Q8h: every 8 hours
RR: reference range
SQ: subcutaneous
TAP: transabdominal plane
tFAST: thoracic focused assessment with sonography for trauma
VCM: viscoelastic coagulation monitoring
WBC: white blood cell

## 1 Introduction

Liver lobe torsion (‘LLT’) is an uncommon condition in veterinary literature that has been identified in dogs, cats, rabbits, horses, sows, otters, amongst others (Schwartz et al. 2006; Swann and Brown 2001; Woolfe and English 1959; Sonnenfield et al. 2001; Von Pfeil et al. 2006; Tomlinson and Black 1983; Weisbroth 1975; Downs et al. 1998; Turner et al. 1993; McConkey et al. 1997; Singh et al. 2002; Hamir 1980; Bhandal et al. 2008; Degner and Bhandal 2013; Lee et al. 2009; Massari et al. 2012; Nazarali et al. 2014; Scheck 2007; Elmasalme et al. 1995; Tubby 2013; Warns‐Petit 2001; Perl et al. 1981). Clinical signs in dogs are non‐specific and can be either acute or chronic in nature (Schwartz et al. 2006; Sonnenfield et al. 2001). The most common clinical signs include abdominal pain, vomiting, lethargy, anorexia, collapse and sudden death. A search of the veterinary literature was performed using terms such as ‘liver lobe’, ‘torsion’ and ‘surgery’. In the literature, dogs with LLT were typically middle to older aged and medium to large breed dogs, with no sex predilection reported (Schwartz et al. 2006; Swann and Brown 2001; Sonnenfield et al. 2001; Degner and Bhandal 2013; Lee et al. 2009; Massari et al. 2012; Nazarali et al. 2014). However, the cause of LLT is still unknown; trauma, stretching or absence of the hepatic ligaments, abnormal anatomy of the liver lobes, entrapment of the liver by the hepatogastric ligament, secondary to gastric dilation and volvulus (‘GDV’) or other gastric surgeries, and hepatic neoplasia have been reported in association with or preceding LLT (Swann and Brown 2001; Sonnenfield et al. 2001; Elmasalme et al. 1995; Tubby 2013; Warns‐Petit 2001).

There have only been a few reported cases of puppies with known LLT (Woolfe and English 1959; Von Pfeil et al. 2006) none of which were an immediate postoperative complication. The purpose of this report is to describe torsion in a puppy of both the left lateral and medial lobes as a postoperative complication, as no underlying causes of the LLT were determined surgically.

## 2 Case Presentation

In July 2023, a 19.8 kg, 6‐month‐old male intact Bernese Mountain Dog was presented to Wheat Ridge Animal Hospital Emergency Service for lethargy and acute vomiting after known foreign body ingestion, as foreign material was found within the vomitus and faeces at home prior to presentation. The morning of presentation, the patient vomited eight socks and became progressively lethargic.

On presentation to the emergency room, the physical exam was unremarkable. A digital rectal exam revealed an empty colon. Three‐view abdominal radiographs (right lateral, left lateral and ventrodorsal projections) were performed and analysed by a board‐certified radiologist. The radiographs revealed gas‐filled small intestines with formed faeces in the colon and suspect partial to fully obstructive foreign material with striations, consistent with cloth material, observed in the small intestines.

As the patient had passed foreign material on his own prior to presentation, overnight hospitalization was recommended. On intake, the patient received ondansetron 0.5 mg/kg IV once and was placed on 75 mL/kg/day of LRS overnight. No food was offered overnight.

**FIGURE 1:**
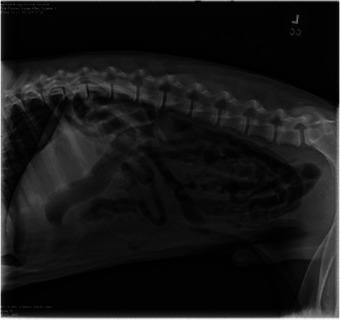
Left lateral abdominal radiograph with a partial to full obstruction of the small intestine by fabric‐like material.

**FIGURE 2:**
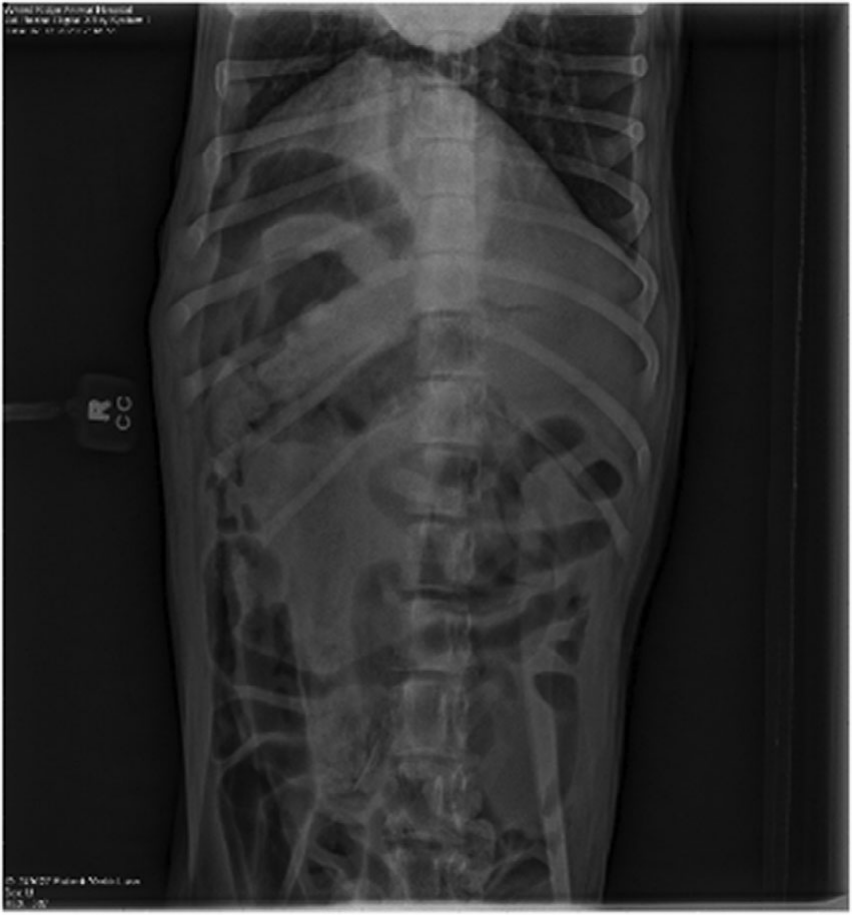
Ventrodorsal abdominal radiograph revealing a partial to full obstruction with a large amount of small intestine affected.

After 12 h of treatment in the hospital, the physical exam was static. Abdominal radiographs (right lateral, left lateral and ventrodorsal projections) were repeated (Figure 1, 2). The finalized radiologist report revealed a persistent partial to full obstruction of the small intestine in the right mid to caudoventral abdomen by fabric‐like material. An exploratory laparotomy was recommended.

Preoperative CBC and chemistry profiles were unremarkable. The patient underwent an exploratory laparotomy. The patient was premedicated with 5 mcg/kg dexmedetomidine IV. An 18‐gauge IV catheter was placed in the right cephalic vein. He was induced with propofol 4 mg/kg IV to effect, midazolam 0.2 mg/kg IV, and lidocaine 2 mg/kg IV. He was maintained under general anaesthesia with isoflurane inhalant, a fentanyl CRI (3mcg/kg/h) and a lidocaine CRI (25 mg/kg/min) and was on LRS 5 mL/kg/h intraoperatively. The patient was intubated with a 47 mm endotracheal tube and placed in dorsal recumbency. His abdomen was shaved and prepped with chlorhexidine using standard operating procedure. He received a TAP block using liposomal bupivacaine at 0.4 mL/kg prior to moving into the operating room. Cefazolin 22 mg/kg IV was given prior to making the skin incision and every 90 min intraoperatively until closure.

A routine exploratory laparotomy via midline celiotomy was performed. A foreign body was present in the mid‐jejunum. The jejunum orad to the foreign material was bruised and dilated. No other abnormalities were detected. An enterotomy was then performed over the most abroad portion of foreign material. The foreign body was removed. The abdomen was closed routinely in 3 layers. The linea alba was closed with 0 PDS in a simple continuous pattern. The subcutaneous fat was opposed with 3/0 Monocryl Plus in a simple continuous pattern. The skin was opposed with 3/0 Monocryl Plus in an intradermal pattern.

Eight hours postoperatively, the patient became tachycardic at 200 bpm and was mildly hypotensive with a mean arterial pressure of 95 mmHg. A Doppler blood pressure was rechecked and was 98 mmHg. POCUS (aFAST) was performed and was negative for free fluid. A tFAST was performed and was negative for free fluid; however, the patient appeared to be underloaded. A fluid bolus of LRS at 10 mg/kg was administered.

Ten hours postoperative, the patient remained tachycardic at 200 bpm. His blood pressure remained static. PCV/TS, blood glucose and lactate were performed and were 34% (reference range: 35%–55%), 4.2 g/dL (RR: 5.5–7.5 g/dL), 128 mg/dL (RR: 65–112 mg/dL) and 2.0 mmol/L (RR: 0.5–2.0 mmol/L), respectively. Hydromorphone 0.05 mg/kg IV was administered due to suspected pain.

Twelve hours postoperative, the patient was reassessed. His mucus membranes were tacky, and an additional 10 mL/kg LRS fluid bolus was administered. The patient became progressively lethargic and unresponsive. The patient's pain was assessed with the Colorado State Canine Acute Pain Scale as a 2 out of 4 on a 4‐point scale. An additional dose of hydromorphone 0.05 mg/kg IV was administered due to suspected abdominal pain. A lidocaine loading dose at 1 mg/kg IV was given, and a lidocaine CRI restarted at 25 mcg/kg/min with hydromorphone 0.05 mg/kg IV q6h PRN.

The patient was persistently tachycardic despite the lidocaine bolus and CRI. The patient progressed to be obtunded, had pale and tacky mucous membranes with thready pulses. Repeat aFAST revealed progressive free fluid. A sample was obtained and was haemorrhagic in appearance. The PCV/TS was 22%, 3.2 g/dL and 25% (RR: 35%–55%), 4.0 g/dL (RR: 5.5–7.5 g/dL) for the abdominal fluid and peripheral blood, respectively.

A CBC, chemistry and blood type were performed. The CBC revealed a leukocytosis characterized by mature neutrophilia with a left shift (WBC 25.5K/uL—RR: 4.4–14.6K/uL, neutrophils 21,607/uL—RR 2394–7514/uL, band cells 510/uL—RR: 0–300/uL) with no other abnormalities. The chemistry revealed hyperphosphatemia (7.7 mg/dL—RR: 2.5–6.8 mg/dL), hypocalcemia (8.3 mg/dL—RR: 9.0–11.0 mg/dL), a panhypoproteinemia (3.4 g/dL—RR: 5.1–6.9 g/dL), and increased ALT (1804 U/L—RR: 17–115 U/L). The patient was blood typed to be DEA 1.1 positive.

A hypertonic saline solution 7.2% USP fluid bolus of 10 mL/kg was administered, followed by 473 mL of DEA 1.1 positive whole blood over 4 h, starting with a 20 mL bolus. Aminocaproic acid 50 mg/kg IV q8h was added to the treatment plan. The patient's mentation improved. Once the blood transfusion was complete, the patient was prepared for surgery.

The patient was premedicated with fentanyl 5 mcg/kg IV and was induced with propofol 4 mg/kg IV to effect. The patient was maintained under general anaesthesia with isoflurane inhalant, fentanyl CRI (3‐5mcg/kg/h) and a lidocaine CRI (25 mg/kg/min). The patient was intubated with a 47 mm endotracheal tube and placed in dorsal recumbency. His abdomen was prepped with chlorhexidine using a standard operating procedure. Cefazolin 22 mg/kg IV was given prior to making the skin incision and every 90 min intraoperatively until closure.

**FIGURE 3:**
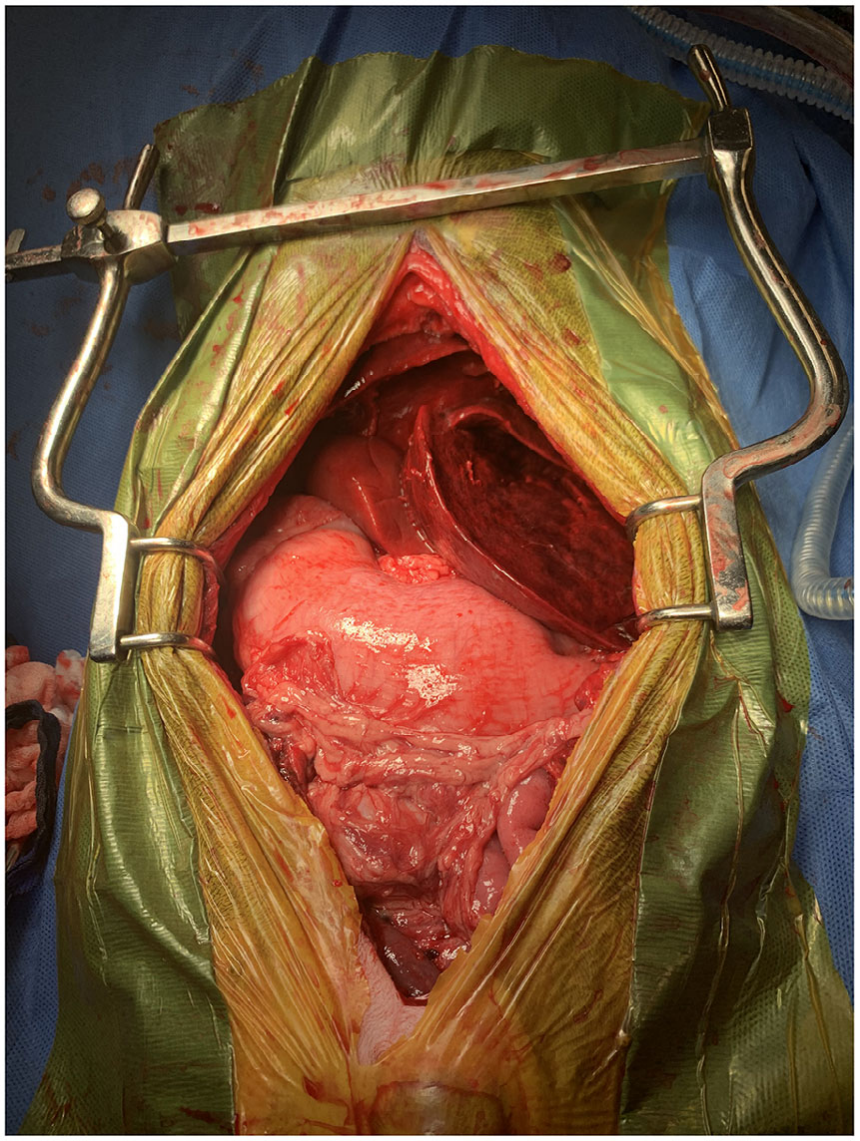
Intraoperative image of the left lateral and left medial liver lobes is torsed, appearing diffusely congested. The cranial aspect of the patient is at the top of the photograph.

**FIGURE 4:**
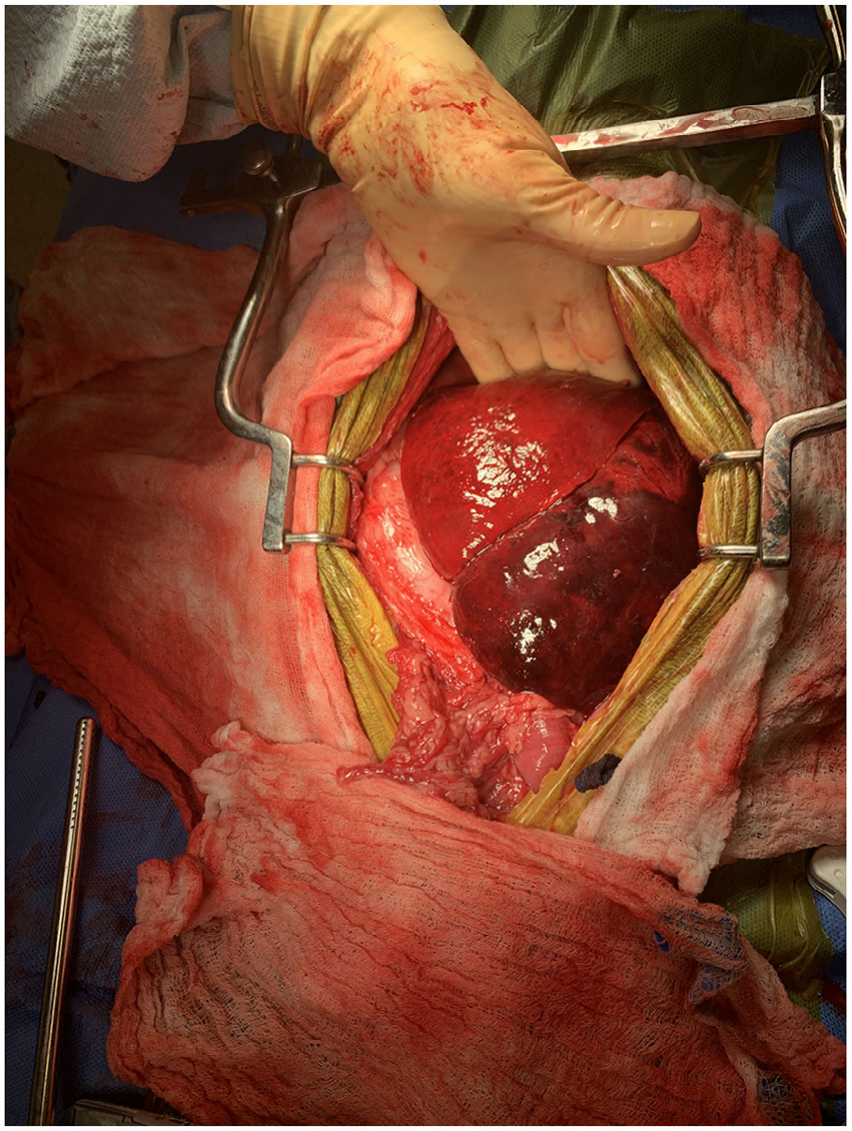
Intraoperative image of the left lateral and left medial liver lobes after the lobes were unrotated. The left lateral liver lobe continued to appear dark and congested. The left medial liver lobe appeared diffusely less congested. The cranial aspect of the patient is at the top of the photograph.

With the patient in dorsal recumbency, another exploratory laparotomy via midline celiotomy was performed. Upon entry approximately 1200 mL of frank blood was observed and suctioned. The left lateral and left medial liver lobes were torsed counterclockwise 180 degrees (Figure 3), appeared diffusely dark red to black and were thick and hard on palpation. The liver lobes were unrotated and the remainder of the abdomen was explored. The remainder of the liver lobes appeared normal. Omental adhesions were observed on the previous intact enterotomy site. The left lateral lobe remained unchanged after the lobe was unrotated. The left medial liver lobe improved in appearance and texture characterized by decrease in dark red patches to bright red and grossly less congested (Figure 4). A thoracoabdominal 30 V‐3 stapler was deployed to perform the left lateral liver lobectomy. A prophylactic right incisional paracostal gastropexy was then performed.

The intestines were then re‐examined. The jejunum appeared dark red to purple with mild diffuse bruising. The mesentery developed diffuse multifocal petechiae. Pulses were observed and palpated through the questionable jejunum and mesentery. Peristalsis was observed through the jejunum. The remainder of the abdomen appeared normal. The abdomen was closed routinely in three layers. The liver lobe was completely submerged in 10% formalin and submitted for histopathology. The patient recovered uneventfully from anaesthesia with no complications noted.

On day 1, post‐op left lateral liver lobectomy and day 2 post‐op enterotomy, the patient improved throughout the day. The patient was noted on physical examination to be non‐painful with a 0/4 as assessed by the Colorado State Canine Acute Pain Scale. Serial aFASTs were static. Recheck PCV/TS was 30% and 4.4 g/dL respectively. His PCV continued to increase throughout the day. Overnight, the patient became mildly hyperthermic, otherwise the physical exam in the evening remained static.

On day 2 post‐op left lateral liver lobectomy and day 3 post‐op enterotomy, the patient's temperature persistently increased. The incision was re‐examined and was painful on palpation, and the cranial aspect of the incision developed a new pocket of edema. Cefazolin 22 mg/kg IV was administered. The patient's temperature normalized soon afterwards.

On day 3 post‐op left lateral liver lobectomy and day 4 post‐op enterotomy, the patient continued to do well. The patient was discharged.

Histologically, the liver lobe revealed severe, acute, massive, lobe infarction and necrosis consistent with acute torsion.

At the 2‐week incision re‐check, the incision was healed, and the patient was reported to be doing well clinically. The patient was approved for gradual return to normal activity.

## 3 Discussion

A search of the veterinary literature reveals that LLT is rare, and there is a lack of information regarding aetiology, diagnosis and signalment (Schwartz et al. 2006). Previous studies do report that the left division of the liver (left lateral and left medial lobes) is more commonly affected in large breed dogs, followed by the caudate, right lateral and lastly right medial lobes (Schwartz et al. 2006; Von Pfeil et al. 2006). This case report is consistent with the left division affected; however, both left medial and left lateral lobes were affected. Double LLT is even more rare, with very few other documented cases (Woolfe and English 1959; Von Pfeil et al. 2006; Bhandal et al. 2008; Massari et al. 2012). One report was in a puppy that was reported to be secondary to aplasia of the left triangular ligament (Woolfe and English 1959). Another was reported in a 5‐month‐old male intact Saint Bernard dog with no previous surgery and no underlying aetiologies reported (Von Pfeil et al. 2006). Another case report documented a 5‐year‐old male neutered Rottweiler with no underlying causes, and another 11‐year‐old male neutered German Shepard with no underlying aetiologies discovered intraoperatively, although the patient underwent an exploratory laparotomy for a splenectomy 4 years prior (Bhandal et al. 2008; Massari et al. 2012). These previously documented cases concluded that more than one liver lobe can be affected; however, there is not always a definitive aetiology to determine the cause of torsion affecting more than one lobe (Woolfe and English 1959; Von Pfeil et al. 2006; Bhandal et al. 2008; Massari et al. 2012). This is consistent with previous reports of unknown aetiologies for LLT in some cases. There have been documented cases with suspected underlying aetiologies for LLT such as trauma, stretching or the absence of the hepatic ligaments, abnormal anatomy of the liver lobes, entrapment of the liver by the hepatogastric ligament, secondary to gastric dilation and volvulus (‘GDV’) or other previous gastric or abdominal surgeries, and hepatic neoplasia have been reported in association with or preceding LLT (Swann and Brown 2001; Sonnenfield et al. 2001; Elmasalme et al. 1995; Tubby 2013; Warns‐Petit 2001). In this case, the patient underwent an exploratory laparotomy for an obstructive jejunal foreign body. At the time of primary surgical intervention, the liver was reported unremarkable with no abnormalities detected. This raises suspicion that previous abdominal surgeries can predispose to LLT and can be a postoperative complication in abdominal surgery.

Although LLT has been reported primarily in mature dogs (Schwartz et al. 2006; Swann and Brown 2001; Tomlinson and Black 1983; Weisbroth 1975; Downs et al. 1998; Turner et al. 1993; McConkey et al. 1997; Singh et al. 2002; Hamir 1980; Bhandal et al. 2008; Degner and Bhandal 2013; Lee et al. 2009; Massari et al. 2012; Nazarali et al. 2014; Scheck 2007; Elmasalme et al. 1995; Tubby 2013; Warns‐Petit 2001; Perl et al. 1981), this report illustrates that regardless of age, torsion of one or more liver lobes should be considered as a differential diagnosis in young dogs with abdominal pain and a haemoabdomen, as well as a postoperative complication for abdominal surgeries. Although there have been previous proposed aetiologies of LLT, the underlying cause of torsion was unknown in this case; however, in this report, the torsion occurred within 12 h postoperatively from a previous abdominal surgical procedure, which included manual manipulation of the liver for evaluation for a standard exploratory laparotomy. Although there have been previous reports of juvenile dogs with LLT, none have been reported as an immediate postoperative complication to the authors’ knowledge. This report describes torsion of both the left lateral and left medial lobes in a young dog as a postsurgical complication with successful management. No underlying cause for these abnormalities could be determined, surgically.

## 4 Conclusion

LLT of the left lateral and medial lobes was found to be an immediate postoperative complication in a 6‐month‐old Bernese Mountain Dog puppy. Clinical signs, results of diagnostics, treatment and outcome have been reported in the past and have all varied significantly (Schwartz et al. 2006; Swann and Brown 2001; Woolfe and English 1959; Sonnenfield et al. 2001; Von Pfeil et al. 2006; Tomlinson and Black 1983; Weisbroth 1975; Downs et al. 1998; Turner et al. 1993; McConkey et al. 1997; Singh et al. 2002; Hamir 1980; Bhandal et al. 2008; Degner and Bhandal 2013; Lee et al. 2009; Massari et al. 2012; Nazarali et al. 2014; Scheck 2007; Elmasalme et al. 1995; Tubby 2013; Warns‐Petit 2001; Perl et al. 1981). Limitations of this report include its retrospective nature and inability to generalize the findings and compare in a larger population. This case report intends to increase the reported case numbers to obtain better data for a better understanding of diagnosing LLT in dogs, as well as introduce LLT, of one or more lobes, as a possible postoperative side effect in any cranial abdominal surgeries.

Clinical signs associated with his LLT included tachycardia, hypotension, haemoabdomen, abdominal pain and dull mentation. Stabilization, resection of the affected liver lobe and postoperative management allowed a successful outcome. LLT has never been reported as an immediate postoperative complication and should be considered as a differential diagnosis in postoperative surgeries in young, large breed dogs with abdominal pain, signs of hypovolemic shock and a haemoabdomen.

## Author Contributions

**Elizabeth Waskover**: conceptualization, data curation, formal analysis, investigation, methodology, project administration, resources, supervision, validation, visualization, writing – original draft, writing – review & editing. **Jennifer Truong**: investigation, writing – original draft. **Lindsay Phillips**: project administration, supervision, visualization, writing – review & editing.

## Ethics Statement

The authors have nothing to report.

## Conflicts of Interest

The authors declare no conflicts of interest.

### Peer Review

The peer review history for this article is available at https://www.webofscience.com/api/gateway/wos/peer‐review/10.1002/vms3.70292.

## Data Availability

The authors have nothing to report.
